# Down-regulation of circular RNA CDC14A peripherally ameliorates brain injury in acute phase of ischemic stroke

**DOI:** 10.1186/s12974-021-02333-6

**Published:** 2021-12-07

**Authors:** Lei Zuo, Jian Xie, Yun Liu, Shuo Leng, Zhijun Zhang, Fuling Yan

**Affiliations:** 1 Department of Neurology, Affiliated ZhongDa Hospital, School of Medicine, Research Institution of Neuropsychiatry, Southeast University, Nanjing, 210009 China; 2 Center of Interventional Radiology and Vascular Surgery, Department of Radiology, Affiliated Zhongda Hospital, Medical School, Southeast University, Nanjing, 210009 China

**Keywords:** Circular RNA CDC14A, Acute ischemic stroke, Neutrophil, Astrocyte activation

## Abstract

**Background:**

Inflammation is integral to the pathophysiology of ischemic stroke and a prime target for the development of new stroke therapies. The aim of the present study is to seek out the regulatory mechanism of circCDC14A in neuroinflammatory injury in tMCAO mice.

**Methods:**

The expression level of circCDC14A in peri-infarct cortex and plasma of mice were detected by qPCR. The localization of circCDC14A in peripheral blood cells and peri-infarct cortex of tMCAO mice were explored by in situ hybridization and immunofluorescence colocalization staining. Lentivirus were microinjected into lateral ventricular of brain or injected into tail vein to interfere with the expression of circCDC14A, thus their effects on behavior, morphology, and molecular biology of tMCAO mice were analyzed.

**Results:**

The expression of circCDC14A in plasma and peri-infarct cortex of tMCAO mice significantly increased, and circCDC14A was mainly localized in neutrophils peripherally while in astrocytes in peri-infarct cortex centrally. Tail vein injection of lentivirus to interfere with the expression of circCDC14A significantly reduced the infarct volume (*P* < 0.01) at 72 h after reperfusion and density of activated astrocytes in peri-infarct cortex at 3 days, 5 days and 7 days after tMCAO modeling (all *P* < 0.0001). Moreover, mNSS (*P* < 0.0001) and survival rate (*P* < 0.001) were significantly improved within 7 days in si-circCDC14A group compared to circCon group. Additionally, morphology analysis showed the volume and surface area of each activated astrocytes significantly decreased (*P* < 0.0001). Quantification analysis measured the percentage of N2 phenotype among infiltrated neutrophils in brain sections and found N2 ratio was significantly higher in si-circCDC14A group compared to circCon group (*P* < 0.001).

**Conclusion:**

Knocking down the expression of circCDC14A in peripheral blood cells relieved astrocytes activation in peri-infarct cortex, thereby relieved brain damage in the acute phase of ischemic stroke.

**Supplementary Information:**

The online version contains supplementary material available at 10.1186/s12974-021-02333-6.

## Background

Stroke is the second leading cause of death in the world and the leading cause of death in China, where a fifth of the world’s population resides [1]. Approximately 70% of all stroke cases, is caused by occlusion of a cerebral artery [2]. In the past few years, recanalization by intravenous (i.v.) thrombolysis and thrombectomy were regarded as first-line treatments for ischemic stroke patients [3]. However, the narrow ‘therapeutic time window’ of recanalization only allows low percent of stroke patients (approximately 10%) benefit from it [4, 5]. Moreover, peripheral immune cells infiltrated into peri-infarct lesion through impaired blood brain barrier (BBB) after stroke, which would further worsen stroke outcome even though recanalization therapy was applied [6, 7]. Post-stroke inflammation indicated brain-resident microglial and astrocytes, infiltrated leukocytes (predominantly polymorphonuclear leukocytes, neutrophils and monocytes/macrophages), as well as cytokines play complex roles causing neuroinflammatory injury after ischemic stroke [7]. Consequently, new therapies targeting fundamental pathogenic mechanisms, especially on post-stroke inflammation, are currently needed experimentally and clinically, either alone or together with recanalization therapy [8].

CircRNAs exert various roles in pathophysiology of ischemic stroke and were prime targets for the development of new stroke therapies [9]. Specifically, knocking down of circHECTD1 leads to the loss of miR-142 sponging, thus depresses TIPARP which resulted in curtailed autophagy of astrocytes and improved post-ischemic functional outcome [10]. Furthermore, overexpression of circDLGAP4 improves the BBB integrity in mice after cerebral ischemia [11]. CircSCMH1 administration significantly improves functional recovery after stroke, with enhancement of neuroplasticity and inhibition of glial reactivity and peripheral immune cell infiltration [12].

Our previous study has verified elevated expression of circCDC14A after stroke, which had potential to be biomarkers in predicting stroke outcome [13]. With the significance evaluated, rationale might be still unclear, and we further explored the mechanism of circCDC14A in acute phase of ischemic stroke. Additionally, our previous study has demonstrated that elevated expression of circCDC14A in plasma mainly caused by the elevation in granulocytes after stroke [13]. Granulocytes, especially neutrophils infiltrated into the brain early after transient middle cerebral artery occlusion (tMCAO) [14], which is considered to be detrimental after stroke, either through the release of neurotoxic proteolytic enzymes [15] or the production of reactive oxygen species (ROS) that contribute to BBB disruption [16]. As the elevated level of circCDC14A in acute phase of stroke has been verified to be associated with stroke outcome, the aim of the present study is to sought out the regulatory mechanism of circCDC14A in neuroinflammatory injury in tMCAO mice.

## Methods

### Animals

Adult male C57BL/6J mice (22.0–24.0 g, 8 weeks old) were purchased from the Model Animal Research Center of Nanjing University (Nanjing, China) and were randomly assigned to experimental groups. Mice were adequately supplied with food and water and housed under a constant temperature with a 12 h light/12 h dark cycle. All animal experiments were approved by the Institutional Animal Care and Use Committee at the Medical School of Southeast University and performed in accordance with the Animal Research: Reporting of In Vivo Experiments (ARRIVE) guidelines.

### tMCAO modeling

Number of mice involved in tMCAO modeling is listed in Additional file 1: Table 1, as well as the number of mice died or failed during or after the procedure. The exact number of mice in different experimental groups was included, too. tMCAO was performed according to a protocol published previously [17] with some adjustments. In brief, anesthesia was induced with 3% isoflurane and maintained with 1.5% isoflurane in 30% oxygen and 70% nitrous oxide using a face mask. After the explosion of the right external carotid artery (ECA), a silicone rubber-coated 6-0 nylon filament (Doccol, Sharon, USA) was inserted from the cut on ECA to the origin of the middle cerebral artery (MCA) along the internal carotid artery (ICA). One hour after the occlusion, the filament was taken out carefully to restore blood flow of the MCA. In sham-operated mice, the ECA was surgically prepared while filament was not inserted.

### RNA isolation and quantitative polymerase chain reaction (qPCR)

Mice subjected to tMCAO were sacrificed at 12 h, 24 h, 3 days, 5 days, 7 days and 9 days after reperfusion. Total RNA was extracted from peri-infarct cortex and plasma of every mouse according to the manufacturer’s protocol [Trizol method and miRNeasy Mini kit (Qiagen)]. Then total RNA was reverse transcribed with the HiScript Q RT SuperMix for qPCR Kit (Vazyme, R123-01) according to the manufacturer’s instructions. Quantitative PCR was performed on the Applied Biosystems QuantStudio 6 (Applied Biosystems) using the manufacturer’s recommended cycling conditions with SYBR Green Real-time PCR Master Mix (Vazyme, R131-01). All samples were run in duplicate. Replicates of individual samples with *Cq* values > 35 were removed from the analysis. The primers used to amplify the circCDC14A transcripts were synthesized by Invitrogen (forward: CTTTGAGACGTTTGATGCGGAA; reverse: GGGTTTGAGCCAGACAGGA). The results were standardized to the control values of GAPDH. Relative expression was calculated using the 2^−ΔΔ*Ct*^ method.

### Lentiviral shRNA vector construction

The plasmids pFU-GW-007-hU6-Ubiquitin-EGFP-IRES-puromycin with circCDC14A siRNA (si-circCDC14A), and their control (circCon) sequences were purchased from Genechem (Shanghai, China) and constructed into lentiviruses. The siRNA sense sequences were as follows: si-circCDC14A, 5′-TATGAACATTATGAGGTCA-3′.

### Microinjection and tail vein injection of circCDC14A siRNA lentivirus

Six-week-old C57BL/6J mice were divided into two groups (circCon and si-circCDC14A) firstly. Then mice were microinjected with either the circControl siRNA-GFP (circCon) lentivirus or circCDC14A siRNA-GFP (si-circCDC14A) lentivirus (2 μL of 1 × 10^9^ TU/mL, Genechem, Shanghai, China) into the left lateral ventricle with a rate of 0.2 μL/min using the following microinjection coordinates: anteroposterior, − 0.3 mm; lateral, 1.0 mm; and ventral, 2.2 mm [18]. Two weeks after microinjection, tMCAO were operated according to protocol described above.

Seven-week-old C57BL/6J mice were divided into two groups (circCon and si-circCDC14A) firstly. Then mice were injected with either circCon lentivirus or si-circCDC14A lentivirus (100 μL of 1 × 10^8^ TU/mL) into tail vein slowly. Seven days after injection, tMCAO were operated according to protocol described above.

### Triphenyl tetrazolium chloride (TTC) staining and cerebral infarction measurement

Infarct volume was evaluated at 72 h after tMCAO. Each brain was coronally sliced into five 1-mm slices with a brain matrix after phosphate buffered saline (PBS) perfusion. Then brain slices were incubated in 2% TTC (Sigma-Aldrich, St. Louis, USA, T8877) at 37 °C for 10 min. Acquired pictures were analyzed with Image J software to evaluate infarct volume. To correct for brain swelling, infarct volume was calculated (contralateral hemisphere volume − ipsilateral non-infarcted volume) by integration of all brain slices.

### Neurological deficit test

Another independent group of tMCAO mice were examined for modified Neurological Severity Score (mNSS). mNSS test was conducted to determine neurological function by a researcher blinded to the experimental groups. At first, mNSS were evaluated 24 h after tMCAO to include qualified mice. Then mNSS test was performed 3, 5 and 7 days after tMCAO by the same researcher who was blinded to the experimental groups. Additionally, mice that died during the tests were recorded carefully.

### Immunostaining and fluorescence in situ hybridization (FISH)

The sections were cut into 30-μm coronal slices by Oscillating slicer (Leica VT1200). The brain sections were subsequently incubated with 0.3% Triton X-100 in PBS for 15 min and blocked with 10% normal goat serum (ZSGB-BIO, ZLI-0956) in 0.3% Triton X-100 for 1 h at room temperature. Next, the sections were incubated with a mouse anti-Ly6g antibody (1:200, abcam, ab25377), rabbit anti-Ym1 antibody (1:400, abcam, ab192029), mouse anti-GFAP antibody (1:500, sigma, G3893), chicken anti-GFAP antibody (1:500, sigma, ab5541), mouse anti-NeuN antibody (1:200, abcam, ab104224) or rabbit anti-Iba-1 antibody (1:200, Wako Pure Chemicals, Osaka, Japan, 019-19741) for colocalization with GFP-positive cells after microinjection or tail vein injection of lentivirus. After being washed with PBS, the sections were incubated with Alexa Fluor 594 goat anti-rabbit IgG (1:250, Invitrogen, Carlsbad, CA, USA, A-11037), Alexa Fluor 594 goat anti-mouse IgG (1:250, Invitrogen, A-11005), or Alexa Fluor 647 goat anti-chicken IgY (1:250, Invitrogen, A-21449) for 1 h. After a final washing step with PBS, brain sections were mounted onto glass slides. Images were captured by microscopy (FV-3000, Olympus, Japan).

FISH in combination with immunostaining fluorescence was performed as described previously [12]. After being permeabilized and prehybridized in hybridization buffer, the brain sections or coverslips were incubated with hybridization buffer containing 50 nM biotin-labeled circCDC14A probes (Invitrogen) at 37 °C for 48 h. After being washed and blocked with a solution of 1% BSA and 3% NGS in PBS for 1 h at room temperature, then brain sections were incubated with FITC-Streptavidin (1:200, Invitrogen, 434311) for 24 h. Afterwards, brain sections were blocked and then incubated with primary antibodies described above overnight at 4 °C. Then, the samples were incubated with secondary antibodies and mounted with Prolong Gold anti-fade reagent containing 4′,6-diamidino-2-phenylindole (DAPI). Immunofluorescence images were captured using confocal microscopy (FV-3000, Olympus, Japan). The mouse circCDC14A probe sequence was 5′-aaaTCTTTAAGTAGATGACCTCATAATGTTCATAT-3′ and was biotinylated at the 5′-end.

### Oxygen glucose deprivation/reperfusion (OGD/R) treatment on primary astrocytes in vitro

Firstly, the primary astrocytes were cultured with deoxygenated DMEM without glucose (Gibco, 11966-025) in an incubator (Thermo Scientific, Waltham, USA) with premixed gas (95% N_2_ and 5% CO_2_) for 3 h and then returned to 95% air, 5% CO_2_, and normal DMEM medium. Control group cultures were cultured with normal DMEM medium for the same incubation time. Astrocytes were subsequently collected before reperfusion or 3 h, 6 h and 12 h after reperfusion.

### Analysis of cell density and morphology

Density and morphology of astrocytes and microglia were assessed using image J software and IMARIS software (IMARIS BITPLANE v.9.0). Integrated densities of neurons were analyzed by image J software. Parameters such as volume or surface area were measured using Imaris Software after creating a 3D surface in the maximum intensity projection image.

### Experimental design and statistical analysis

Statistical analysis was performed by GraphPad Prism 8 Software. Data are expressed as mean ± standard error of mean (SEM) and *P* < 0.05 was considered to indicate significance. Shapiro–Wilk tests were used to assess the normality of the distribution for each group. Significance was assessed with Student’s *t*-test (two-tailed) or the Mann–Whitney *U* test for continuous variables. One- or two-way analysis of variance (ANOVA) followed by Holm–Sidak tests was used for comparisons of 3 or more groups. Log rank test was used in Kaplan–Meier curve in assessing survival rate.

## Results

### Gene information and temporal expression of circCDC14A in plasma and peri-infarct cortex of tMCAO mice

CircCDC14A is made by reverse splicing of exons 5, 6 and 7 on gene CDC14A (Additional file 1: Fig. 1a). Species conservation analysis showed that gene sequences that make up circCDC14A have high interspecies conservation between humans and mice, the homology of which is 90%. Furthermore, compared to heart, liver and spleen, the expression of circCDC14A is relatively higher in brain, lung and kidney of mice(Additional file 1: Fig. 1b).

**Fig. 1:**
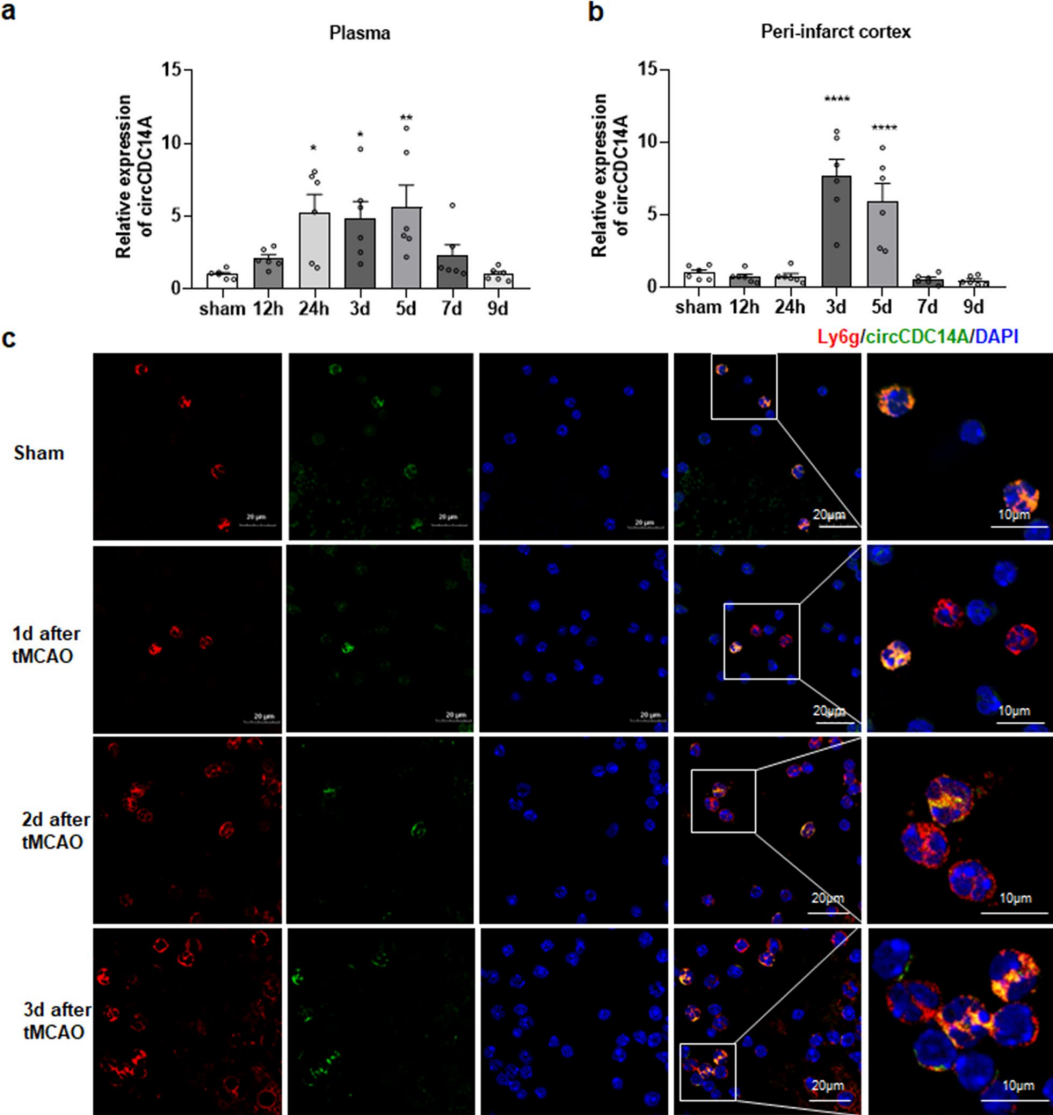
Temporal expression profile of circCDC14A in plasma and peri-infarct cortex of tMCAO mice and colocalization of circCDC14A with neutrophils. **a** The change of circCDC14A in plasma from 12 h to 9 days after stroke, *n* = 6 mice per group. *P < 0.05, **P < 0.01, one-way ANOVA test. **b** The change of circCDC14A in peri-infarct cortex from 12 h to 9 days after stroke, *n* = 6 mice per group. ****P < 0.0001, one-way ANOVA test. **c** Colocalization of circCDC14A with neutrophils in sham-operated mice and tMCAO mice 1 day, 2 days and 3 days after modeling

Afterwards, expression levels of circCDC14A in plasma and peri-infarct cortex of tMCAO mice were measured up to 9 days after tMCAO. The level of circCDC14A in plasma showed an upward trend within 12 h and significantly increased by 24 h after reperfusion. The significant increased level of circCDC14A continued until to the 5th day and dropped to normal level in the 7th day after tMCAO (Fig. 1a). However, the level of circCDC14A in peri-infarct cortex significantly increased from 3 days after tMCAO modeling, while returned to normal at 7 days after tMCAO. The temporal inconsistency of the change trend indicated that peripheral circCDC14A increased prior to that in peri-infarct cortex (Fig. 1b).

### CircCDC14A elevated level is mainly derived from neutrophils peripherally in tMCAO mice

By colocalization of FISH and immunofluorescence staining of peripheral neutrophil surface marker Ly6g and circCDC14A probes, it was found that circCDC14A is mainly localized in neutrophils and differentially expressed in various neutrophil populations (Fig. 1c). The same colocalization was observed in peripheral neutrophils from the 2nd day to the 3rd day after tMCAO modeling (Fig. 1c).

### The elevation of circCDC14A levels in peri-infarct cortex of tMCAO mice mainly localized in astrocytes

**Fig. 2:**
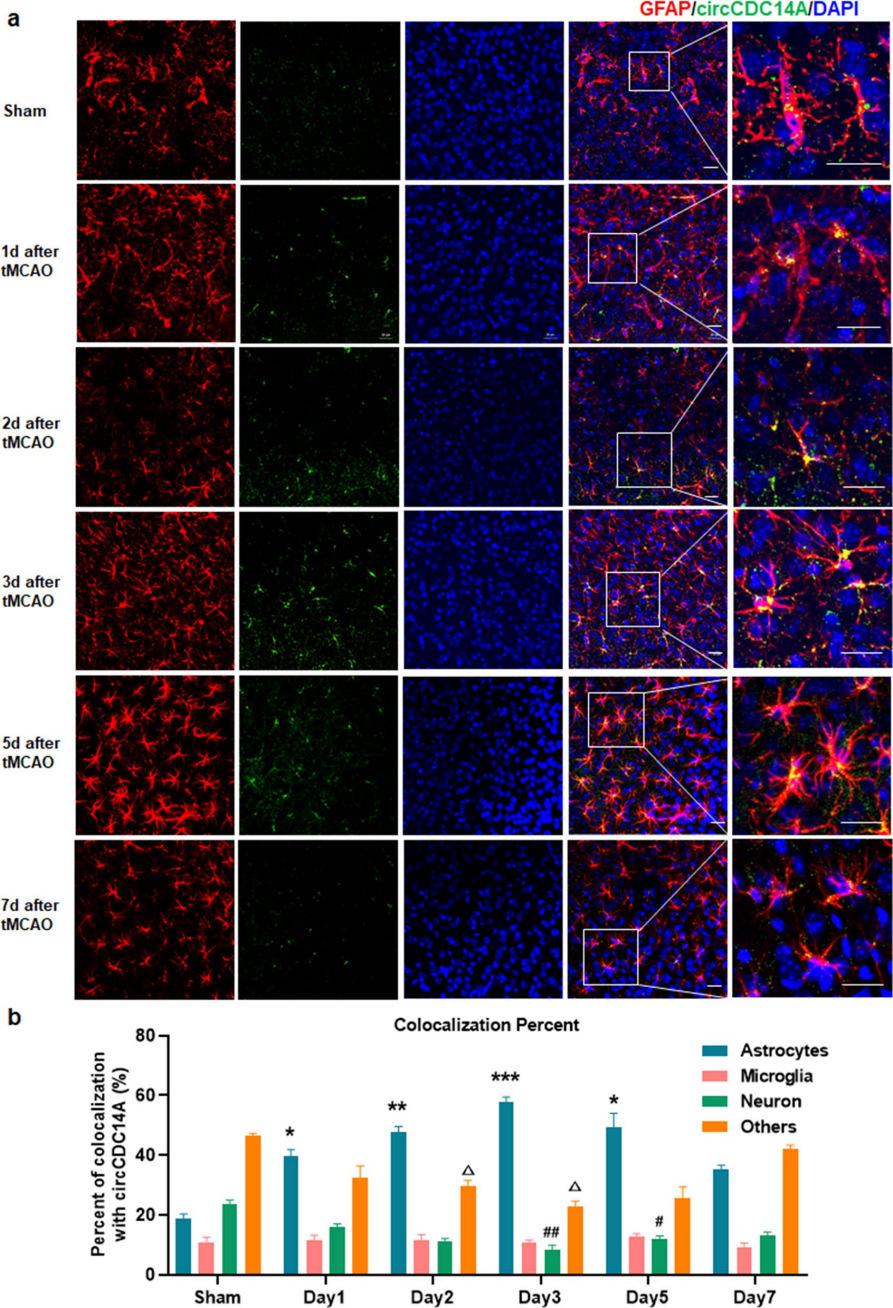
Colocalization analysis of circCDC14A with astrocytes, microglia, neuron and others in peri-infarct cortex. **a** Colocalization with GFAP in sham-operated and tMCAO mice from 1 to 7 days after modeling. Bar: 20 μm. **b** Quantification of circCDC14A colocalization percent with astrocytes, microglia, neuron and others, *n* = 3 mice per group. *P < 0.05, **P < 0.01, ***P < 0.001, compared to astrocytes in sham-operated mice; ^#^*P* < 0.05, ^##^*P* < 0.01, compared to neuron in sham-operated mice; ^∆^*P* < 0.05, compared to others in sham-operated mice; two-way ANOVA test

Afterwards, we further explored cell distribution of elevated circCDC14A in peri-infarct cortex of tMCAO mice. Firstly, colocalization of circCDC14A in sham-operated mice and tMCAO mice revealed that the elevated circCDC14A in peri-infarct cortex of tMCAO mice was mainly colocalized with astrocytes (Fig. 2a), while no significant colocalization was observed in neuron (Additional file 1: Fig. 2) and microglia (Additional file 1: Fig. 3). Furthermore, quantification analysis showed the colocalization with astrocytes significantly increased from 1 to 5 days after tMCAO modeling compared to sham-operated mice, while dropped to normal level in 7 days after modeling (Fig. 2b). In contrast, the colocalization with neurons and others significantly decreased in 2 days and 3 days, respectively, compared to sham-operated mice (Fig. 2b). No significant change was observed in colocalization with microglia within 7 days after tMCAO modeling compared to sham-operated mice (Fig. 2b).

**Fig. 3:**
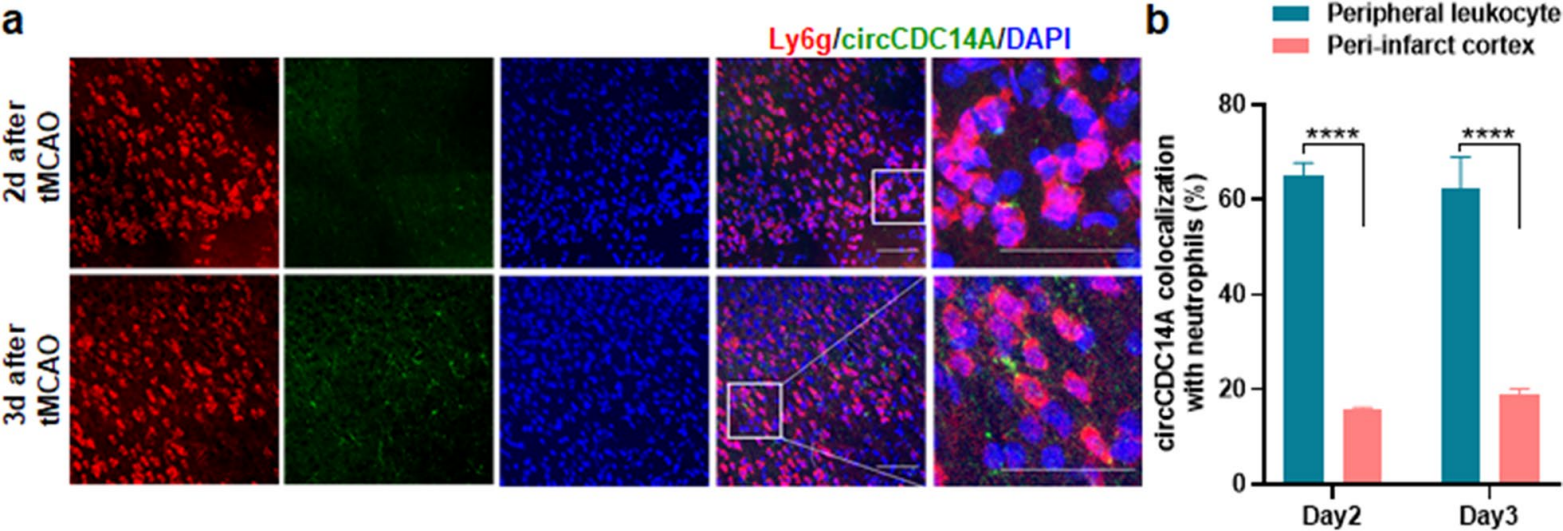
Colocalization of circCDC14A with neutrophils. **a** Colocalization of circCDC14A with neutrophils in peri-infarct cortex of tMCAO mice from 2 to 3 days after modeling. Bar: 50 μm. **b** Quantification of circCDC14A colocalization with neutrophil in peri-infarct cortex and peripheral leukocytes

In addition, neutrophils were found in infarct cortex 2 and 3 days after reperfusion, that were colocalized with circCDC14A probes (Fig. 3a). However, for the neutrophils that infiltrated into infarct cortex, the colocalization with circCDC14A is significantly decreased than that with peripheral neutrophils (Fig. 3b).

### Origins of elevated circCDC14A in astrocytes of peri-infarct cortex

**Fig. 4:**
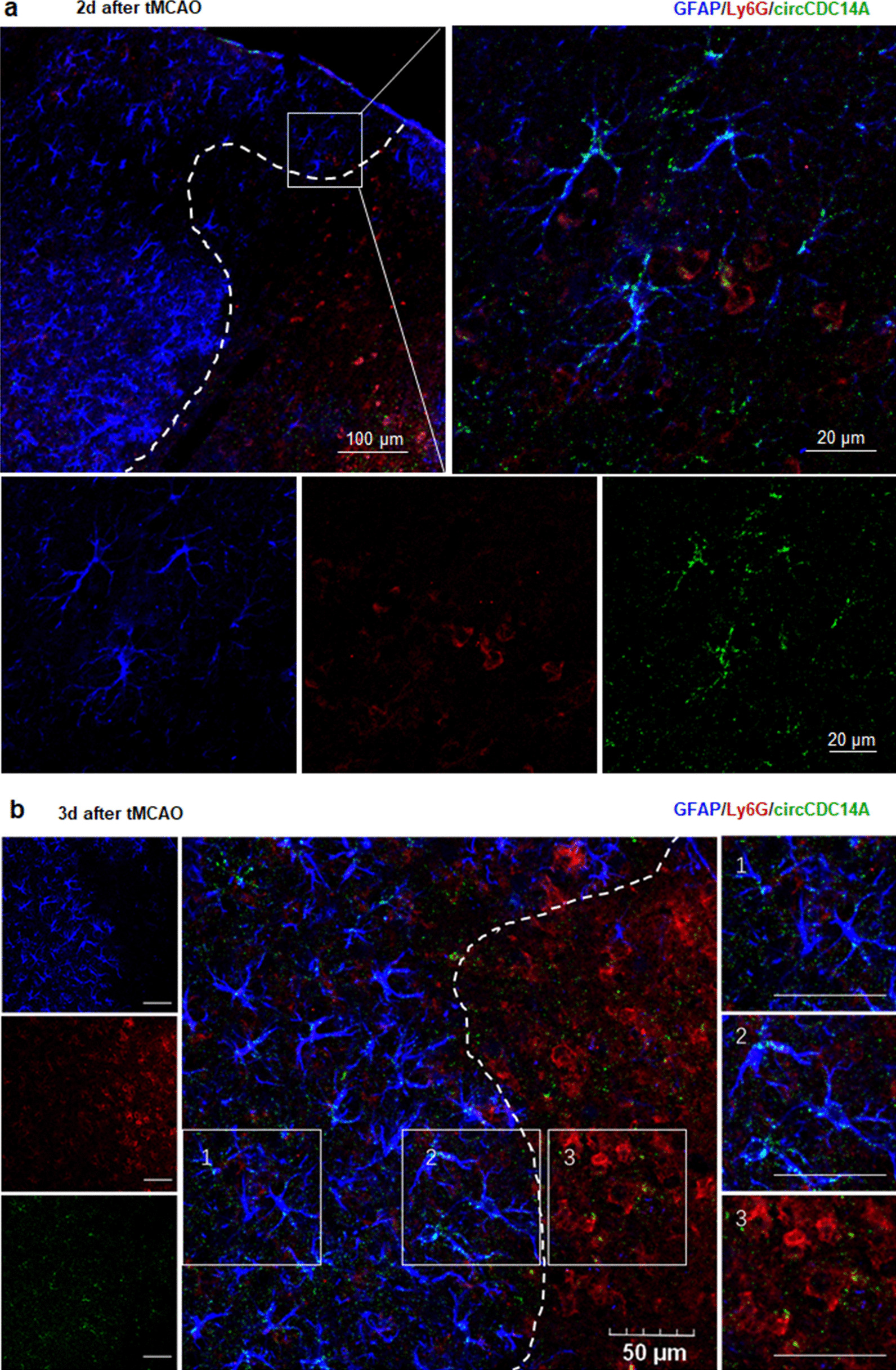
Colocalization of circCDC14A with astrocytes together with neutrophils in tMCAO mice 2 days and 3 days after modeling. **a** Colocalization with GFAP together with Ly6g in tMCAO mice 2 days after modeling, bar: 50 μm. Dashed line indicated the border of the infarct lesion. **b** Colocalization with GFAP together with Ly6g in tMCAO mice 3 days after modeling

Further, we want to figure out the interaction of peripheral and central elevated cirCDC14A. Two days after tMCAO modeling, according to colocalization of circCDC14A in situ hybridization with immunofluorescent staining of astrocytes and neutrophils surface markers, significant colocalizations were found in neutrophils and their surrounding astrocytes (Fig. 4a). Three days after tMCAO modeling, the closer astrocyte was to the neutrophils infiltrated into the brain, the more circCDC14A were colocalized with astrocytes. No significant circCDC14A colocalization was found in astrocytes in peri-infarct cortex where without infiltrated neutrophils (Fig. 4b). Therefore, infiltrated neutrophils might be important origins of elevated circCDC14A in astrocytes.

However, circCDC14A in astrocytes might be increased endogenously because of oxygen–glucose deprivation after stroke. Next, oxygen–glucose deprivation/reoxygenation (OGD/R) on mouse primary astrocytes showed that the level of circCDC14A were not higher than that of the control group within 12 h after reperfusion (Additional file 1: Fig. 4), not as the upward trend as in vivo experiments, indicating that the elevation of circCDC14A in astrocytes was not caused by ischemic damage, but might be originated from infiltrated neutrophils.

### Knocking down the expression of circCDC14A in brain cortex by microinjection of lentivirus

**Fig. 5:**
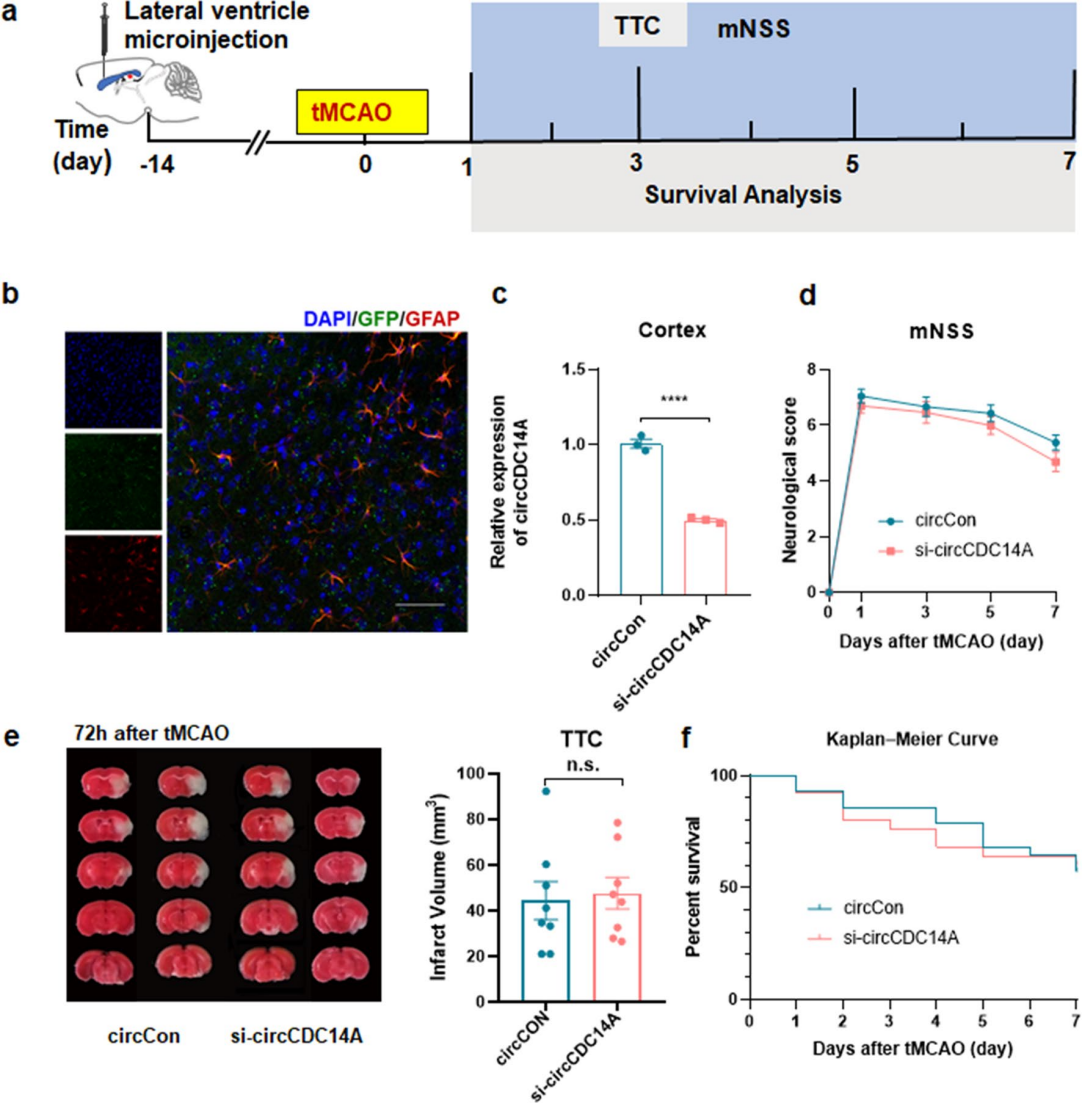
Knocking down the expression of circCDC14A in cortex. **a** Flow chart of the experiment design. **b** Colocalization of GFP lentivirus with GFAP 14 days after microinjection. Bar: 50 μm. **c** Expression of circCDC14A in cortex, *n* = 3 mice per group. ****P < 0.0001, Student’s *t* test. **d** Neurological deficit score of tMCAO mice in 1 day, 3 days, 5 days and 7 days after modeling. CircCon *n* = 18, si-circCDC14A *n* = 17. No significance, two-way ANOVA test. **e** Measurement of infarct volume by TTC staining 72 h after tMCAO, *n* = 8 mice per group. No significance, Student’s *t* test. **f** Kaplan–Meier curve of tMCAO mice in 7 days after modeling. CircCon *n* = 28, si-circCDC14A *n* = 25. No significance, log rank test

Next, lateral ventricle microinjection of lentivirus was performed 2 weeks before tMCAO modeling to knock down the expression of circCDC14A in brain cortex (Fig. 5a). As shown in the figure, 2 weeks after microinjection, immunofluorescence staining revealed significant viral fluorescence expression in the cortex which had colocalization with astrocytes (Fig. 5b). Furthermore, the expression of circCDC14A in mice injected with circCDC14A knockdown lentivirus was significantly reduced, and the knockdown efficiency could reach 50% (Fig. 5c). Later on, mNSS of tMCAO mice were evaluated at 1 day, 3 days, 5 days and 7 days after modeling, shown that knocking down the expression of circCDC14A in cortex had no significant effect on the neurological deficit scores of tMCAO mice (Fig. 5d). Besides, Infarct volume of the tMCAO mice was evaluated by TTC staining 72 h after reperfusion, found no significant difference between two groups (Fig. 5e). Moreover, no significant difference was calculated in the survival rate of tMCAO mice between two groups within 7 days after modeling (Fig. 5f).

### Knocking down expression of circCDC14A in peripheral blood cells by tail vein injection of lentivirus

**Fig. 6:**
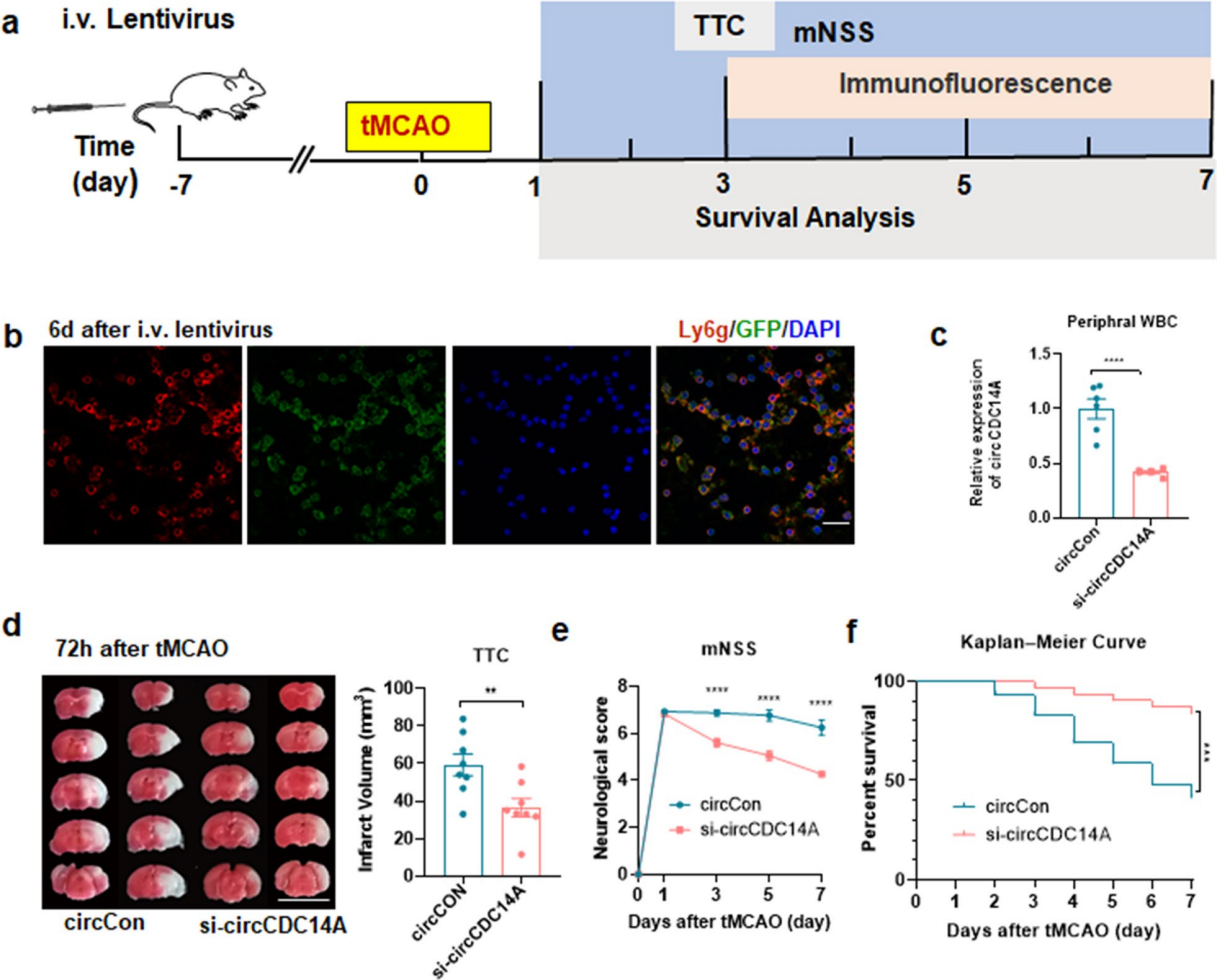
Knocking down the expression of circCDC14A in peripheral blood cells. **a** Flow chart of the experiment design. **b** Colocalization of GFP lentivirus with Ly6g 6 days after tail vein injection. Bar: 20 μm. **c** Expression of circCDC14A in peripheral white blood cells, *n* = 6 mice per group. ****P < 0.0001, Student’s *t* test. **d** Measurement of infarct volume by TTC staining on 72 h after tMCAO, *n* = 8 mice per group. **P < 0.01, Student’s *t* test. **e** Neurological deficit score of tMCAO mice in 1 day, 3 days, 5 days and 7 days after modeling. CircCon *n* = 16, si-circCDC14A *n* = 18. ****P < 0.0001, two-way ANOVA test. **f** Kaplan–Meier curve of tMCAO mice in 7 days after modeling. CircCon *n* = 25, si-circCDC14A *n* = 25. ***P < 0.001, log rank test

The flowchart of knocking down the expression of circCDC14A in peripheral blood cells is shown in Fig. 6a. Significant and uniform expression of viral fluorescence were observed in peripheral blood leukocytes 6 days after tail vein injection of lentivirus and were colocalized with neutrophils (Fig. 6b). The expression of circCDC14A in peripheral white blood cells significantly decreased examined by qPCR, and the knockdown efficiency could reach more than 50% (Fig. 6c), while the mRNA level of CDC14A was not inhibited (Additional file 1: Fig. 5). Therefore, tMCAO modeling was conducted 7 days after the tail vein injection of lentivirus. TTC staining was performed 72 h after reperfusion to evaluate the infarct volume of tMCAO mice, suggesting that knocking down the expression of circCDC14A peripherally significantly reduced infarct volume in tMCAO mice (Fig. 6d). Neurological deficit scores were evaluated on 1 day, 3 days, 5 days and 7 days after tMCAO, found that there was no significant difference in the mNSS at 1 day after modeling, while the mNSS scores were significantly lower in tMCAO mice injected with si-circCDC14A lentivirus compared to circCon group on 3 days, 5 days and 7 days after modeling (Fig. 6e). Besides, survival rate of si-circCDC14A group were significantly higher than circCon group within 7 days after tMCAO modeling (Fig. 6f).

### Knockdown of circCDC14A expression in peripheral blood cells inhibit astrocytes activation in peri-infarct cortex

**Fig. 7:**
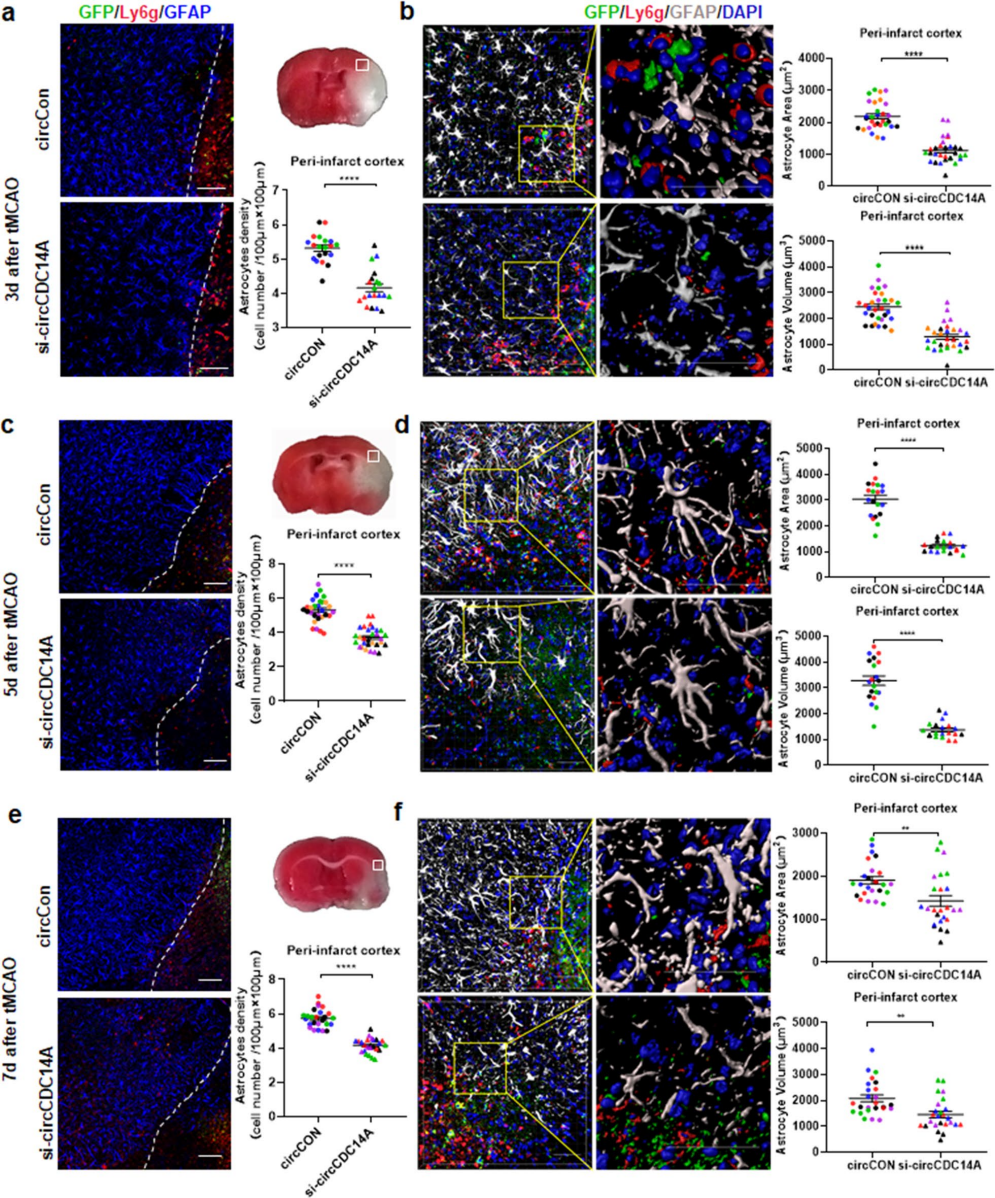
Activation of astrocytes in peri-infarct cortex of tMCAO mice in 7 days after modeling. **a** Representative images of immunofluorescence showing activated astrocytes (GFAP) and neutrophils (Ly6g) in peri-infarct cortex 3 days after modeling. Dashed line showed the border of the infarct lesion. Bar: 100 μm. Density of activated astrocytes in peri-infarct cortex were analyzed, *n* = 5 field (200 μm × 200 μm) *6 different mice represented by different colors per group. ****P < 0.0001, Student’s *t* test. **b** 3D-reconstruction (Imaris) of a representative astrocyte cell per region 3 days after modeling. Areas and volumes of activated astrocytes in peri-infarct cortex were analyzed by Imaris software, *n* = 5 astrocytes (200 μm × 200 μm) *6 different mice represented by different colors per group. ****P < 0.0001, Student’s *t* test. **c** Representative images of immunofluorescence showing activated astrocytes (GFAP) and neutrophils (Ly6g) in peri-infarct cortex 5 days after modeling. Bar: 100 μm. Density of activated astrocytes in peri-infarct cortex were analyzed, *n* = 5 field (200 μm × 200 μm) *4 different mice represented by different colors per group. ****P < 0.0001, Student’s *t* test. **d** 3D-reconstruction (Imaris) of a representative astrocyte cell per region 5 days after modeling. Areas and volumes of activated astrocytes in peri-infarct cortex were analyzed by Imaris software, *n* = 5 field (200 μm × 200 μm) *4 different mice represented by different colors per group. ****P < 0.0001, Student’s *t* test. **e** Representative images of immunofluorescence showing activated astrocytes (GFAP) and neutrophils (Ly6g) in peri-infarct cortex 7 days after modeling. Bar: 100 μm. Density of activated astrocytes in peri-infarct cortex were analyzed, *n* = 5 field (200 μm × 200 μm) *5 different mice represented by different colors per group. ****P < 0.0001, Student’s *t* test. **f** 3D-reconstruction (Imaris) of a representative astrocyte cell per region 7 days after modeling. Areas and volumes of activated astrocytes in peri-infarct cortex were analyzed by Imaris software, *n* = 5 field (200 μm × 200 μm) *5 different mice represented by different colors per group. **P < 0.01, Student’s *t* test

Knocking down the expression of circCDC14A in peripheral blood cells significantly relieved the activation of astrocytes in peri-infarct cortex. Specifically, cell density of activated astrocytes in peri-infarct cortex significantly decreased in si-circCDC14A group compared to circCon group at 3 days, 5 days and 7 days after modeling (Fig. 7a, c and e). Moreover, 3D-reconstructions based on Z-stack slices were conducted by Imaris software. Morphological analysis of the astrocytes in peri-infarct cortex demonstrated that the surface area and volume (Fig. 7b, d and f) of the astrocytes were significantly lower in tMCAO mice injected with circCDC14A knockdown lentivirus compared to those injected with circCon lentivirus.

Microglia and cytokines were evaluated to further examine neuroinflammation state after stroke. We stained microglia with anti-iba-1 antibodies in peri-infarct cortex (Additional file 1: Fig. 6a) and found cell density of activated microglia were not significant different between two groups at 3 days after modeling (Additional file 1: Fig. 6b). Moreover, 3D-reconstructions of microglia based on Z-stack slices in peri-infarct cortex demonstrated that no difference was found in surface area and volume of the microglia (Additional file 1: Fig. 6c, d) between two groups. Additionally, the integrated density of neuron in these two groups were not significant different, too (Additional file 1: Fig. 6e). Iba-1 proteins from peri-infarct cortex were detected 3 days after reperfusion, and no significant difference was observed between two groups, too (Additional file 1: Fig. 6f).

To investigate the effect of circCDC14A silencing on pro-inflammatory and anti-inflammatory cytokine secretion, the contents of tumor necrosis factor-α (TNF-α), interleukin (IL)-10 and IL-6 in peri-infarct cortex were detected at 3 days after modeling using ELISA. The levels of TNF-α, IL-6, and IL-10 in si-circCDC14A group were not significant different from those in circCon group (Additional file 1: Fig. 6g–i), indicating pro-inflammatory and anti-inflammatory cytokine secretion was not suppressed or stimulated.

### Inhibiting circCDC14A expression peripherally modulates neutrophil polarization

**Fig. 8:**
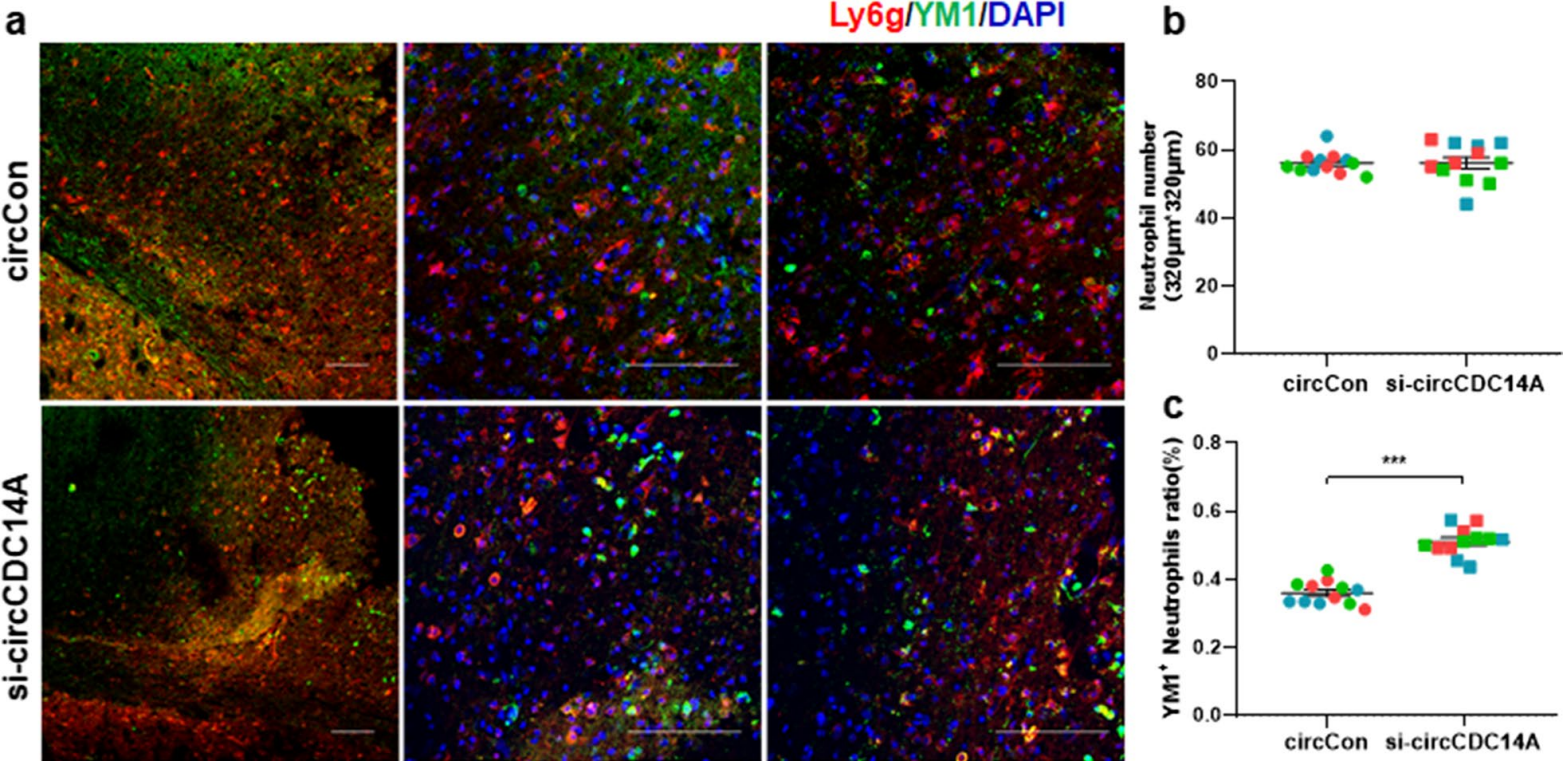
Inhibiting circCDC14A expression peripherally modulates neutrophil polarization. **a** Representative photomicrographs showing YM1 (green) and Ly6g (red) double immunostaining after tMCAO in circCon (top) or si-circCDC14A mice (bottom). Bar: 100 μm. **b** Quantification of Ly6g^+^ neutrophils in two groups, *n* = 2 field (320 μm × 320 μm) *3 different mice represented by different colors per group, no significance, Student’s *t* test. **c** N2 neutrophils (YM1^+^, Ly6g^+^) ratio in peri-infarct cortex of both groups. *n* = 2 field (320 μm × 320 μm) *3 different mice represented by different colors per group, ***P < 0.001, Student’s *t* test

Infiltrated total neutrophils were analyzed first to explore whether knockdown of circCDC14A expression in peripheral blood cells modulates neutrophil infiltration, while no significant difference was found in two groups (Fig. 8a, b). Furthermore, previous research has described a population of neutrophils, so-called N2, that expressed chitinase-like protein (also named YM1) in experimental ischemia [19]. Thus, we selected this marker to explore whether knocking down circCDC14A peripherally affects the relative proportions of N2 neutrophil populations in the ischemic brain. Quantification analysis measured the percentage of N2 (Ly6G^+^/YM1^+^) phenotype among infiltrated neutrophils in brain sections and found N2 ratio was significantly higher in si-circCDC14A group compared to circCon group (Fig. 8a, c, *P* < 0.001).

## Discussion

**Fig. 9:**
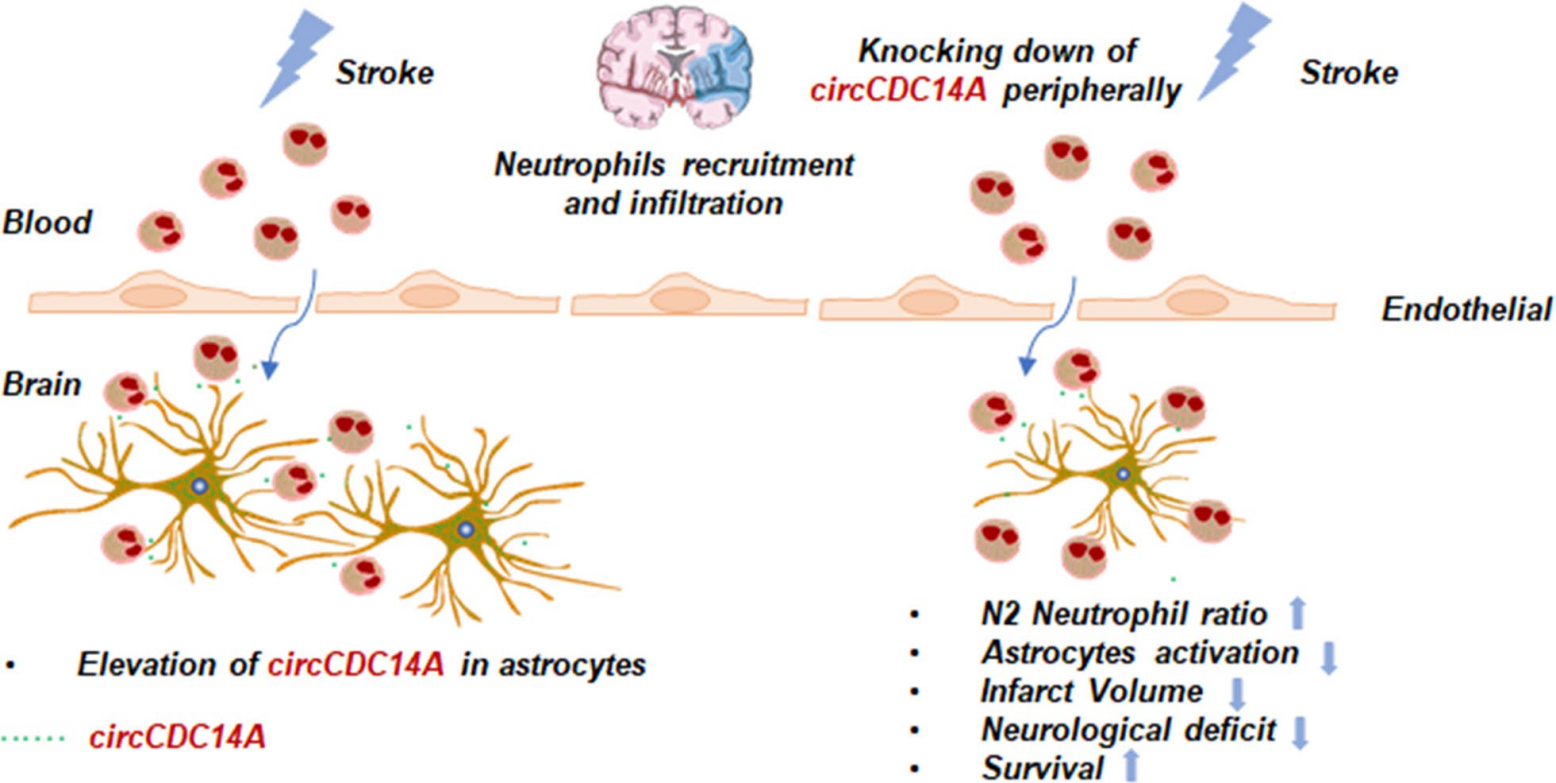
The proposed regulation way of circCDC14A in acute phase of stroke. After tMCAO modeling, the elevation of circCDC14A in plasma was prior to that in peri-infarct cortex. Elevated circCDC14A were mainly located in neutrophils peripherally and astrocytes centrally. Through the impaired blood–brain barrier, neutrophils were important medium for peripheral and central delivery of circCDC14A. Knocking down the expression of circCDC14A peripherally changed the phenotype of infiltrated neutrophils from N1 to N2 and relieved astrocytes activation, which helps to decrease infarct volume, neurological deficit and increase survival rate in acute phase of tMCAO modeling

Our previous research has confirmed the significantly elevated circCDC14A level in plasma of acute ischemic stroke (AIS) patients, which could be a biomarker for acute ischemic stroke in diagnosis and predicting prognosis. Therefore, a series of experiments were conducted in tMCAO mice to further explore the mechanism of how elevated circCDC14A endanger the prognosis of stroke. Firstly, we found that the level of circCDC14A significantly increased either in plasma or in peri-infarct cortex of tMCAO mice, and the elevation in plasma was prior to that in peri-infarct cortex. Afterwards, we have confirmed that the elevated circCDC14A were mainly located in neutrophils peripherally and in astrocytes centrally. Through the impaired blood–brain barrier, neutrophils were important medium for peripheral and central inflammatory regulation, exerting the role of circCDC14A in regulating inflammatory injury (Fig. 9) and providing a theoretical basis for its potential role as a therapeutic target in AIS.

Several studies have shown that circRNAs were involved in inflammatory regulation, especially neuroinflammatory regulation, thereby regulating inflammatory injury and stroke progression [20–22]. Previous research has demonstrated that circHIPK2 can participate in the neuroinflammatory response caused by lipopolysaccharide (LPS) [20]. In addition, circHECW2 is involved in the BBB damage caused by LPS and methamphetamine [21]. In tMCAO mice, overexpression of circDLGAP4 improves the BBB integrity in mice after cerebral ischemia [11]. CircSCMH1 administration significantly improves functional recovery after stroke, with enhancement of neuroplasticity and inhibition of glial reactivity and peripheral immune cell infiltration [12]. All the above research illustrated the role of circRNAs in physiology and pathophysiology regulations, indicated their potential application in stroke therapies.

Neutrophils affect the occurrence, progression and prognosis of acute ischemic stroke through various biological processes [23]. Matrix metalloproteinase-9 [24] and myeloperoxidase [25] which considered as mediators of the neutrophil effects, and other potential mediators such as elastase [26] and leukotriene B4 [27] derived from neutrophils need to be evaluated in following research to verify the correlation with circCDC14A after stroke. Infiltration of neutrophils into infarct cortex was proposed to be main origins of circCDC14A in peri-infarct cortex, which delivered to astrocytes and exerting its role in inflammatory damage after stroke. The specific elevation of circCDC14A in astrocytes proposed further assumptions for our research. Astrocytes are important neuroinflammatory regulatory cells in the brain. Previous studies have proved that circHECTD1 mainly aggravates neuroinflammatory damage by regulating the autophagy of astrocytes, illustrated that circHECTD1 had the potential to be an effective therapeutic target for acute ischemic stroke. Therefore, the downstream mechanism of how circCDC14A regulates astrocytes activation need to be further explored.

As mentioned above, post-stroke neuroinflammation is an imperative target for stroke treatment. The present study focused on the peripheral neutrophils and central astrocytes and evaluated the effect of knocking down circCDC14A either peripherally or centrally. Though peripheral interference of circCDC14A ameliorated brain injury in acute phase of ischemic stroke, the interference was not selective which need further investigations on selective targeting neutrophils. Another important shortcoming of the present study is that we could not exclude the elevation of circCDC14A in astrocytes was endogenous. However, the synthesis efficiency of circular RNA in vitro is very low, which made marking and tracing of circular RNA difficult currently. Moreover, the results of OGD/R on primary astrocytes illustrated that the elevation of circCDC14A was not caused by ischemia damage, further verified exogenous increase of circCDC14A in peri-infarct cortex.

In the past, there have been many attempts to treat stroke by intervention on the peripheral immune system, mainly through the intervention of cytokine receptors, such as TNF-α, IL-1, IL-6 and IL-10 [9], while most of the attempts failed to be translated into clinical application [9]. In the present study, knocking down the expression of circCDC14A peripherally changed the phenotype of infiltrated neutrophils from N1 to N2 and suppressed astrocyte activation which provide new hope for peripheral intervention treatment. For anti-inflammatory therapy to be successful in stroke treatment, it is necessary to understand the spatiotemporal dynamics of inflammatory glial cells in the brain, inflammatory cells recruited into the brain, and peripheral immune cells [28]. The most advanced single-cell sequencing technology for studying cell subtype could be used to determine astrocytes and neutrophils infiltrating into the brain, which is a very promising preclinical study to prove the mechanism of circCDC14A in specific neutrophil subtypes on neuroinflammatory injury and a hot spot for future stroke research. Generally, it requires further translational studies to target specific neutrophils subtypes in stroke treatment.

As reperfusion therapy has been widely acknowledged, more attempts should focus on reperfusion injury, especially for neuroinflammation. The result of the present study found neutrophil-derived circCDC14A was critical for astrocytes activation, which worsen stroke outcome. Therefore, targeting neutrophil to interfere with circCDC14A expression peripherally might be an accessible way to relieve neuroinflammatory injury, combined with reperfusion therapy (intravenous thrombolysis and thrombectomy). Though more molecular mechanism might be explored and verified in the following research.

## Conclusions

Knocking down the expression of circCDC14A in peripheral blood cells relieved astrocytes activation after stroke, thereby relieved brain damage in the acute phase of ischemic stroke. Peripheral interference with the expression of circCDC14A is expected to be an effective treatment for brain injury after acute ischemic stroke.

## Supplementary Information

**Additional file 1: Table S1.** Involved experimental mice in tMCAO modeling. **Figure S1.** Gene information and organ distribution in mice of circCDC14A. **Figure S2.** Colocalization of circCDC14A with neuron in peri-infarct cortex of sham-operated and tMCAO mice from 1 day up to 7 days after modeling. **Figure S3.** Colocalization of circCDC14A with microglia in peri-infarct cortex of sham-operated and tMCAO mice from 1 day up to 7 days after modeling. **Figure S4.** Relative expression of circCDC14A in OGD/R treated primary astrocytes. **Figure S5.** The effect of knocking down circCDC14A peripherally on mRNA level of CDC14A in peripheral WBC and peri-infarct cortex. **Figure S6.** The effect of knocking down circCDC14A peripherally on neuroinflammation state of microglia and cytokines in tMCAO mice.

## Data Availability

The data that support the findings of this study are available from the corresponding author upon reasonable request.

## Abbreviations

AIS: Acute ischemic stroke
ANOVA: Analysis of variance
BBB: Blood–brain barrier
DAPI: 4',6-Diamidino-2-phenylindole
ECA: External carotid artery
FISH: Fluorescence in situ hybridization
ICA: Internal carotid artery
IL: Interleukin
LPS: Lipopolysaccharide
MCA: Middle cerebral artery
mNSS: Modified neurological severity score
OGD/R: Oxygen–glucose deprivation/reoxygenation
PBS: Phosphate buffered saline
qPCR: Quantitative polymerase chain reaction
ROS: Reactive oxygen species
SEM: Standard error of mean
tMCAO: Transient middle cerebral artery occlusion
TNF-α: Tumor necrosis factor-α
TTC: 2,3,5-Triphenyle tetrazolium chloride
