# Supplementary material for: Down-regulation of circular RNA CDC14A peripherally ameliorates brain injury in acute phase of ischemic stroke
Source: J Neuroinflammation. 2021 Dec 7;18:283. doi: 10.1186/s12974-021-02333-6 (PMC8653620; doi:10.1186/s12974-021-02333-6)
Supplement: Supplementary file 1 — Additional file 1: Table S1. Involved experimental mice in tMCAO modeling. Figure S1. Gene information and organ distribution in mice of circCDC14A. Figure S2. Colocalization of circCDC14A with neuron in peri-infarct cortex of sham-operated and tMCAO mice from 1 day up to 7 days after modeling. Figure S3. Colocalization of circCDC14A with microglia in peri-infarct cortex of sham-operated and tMCAO mice from 1 day up to 7 days after modeling. Figure S4. Relative expression of circCDC14A in OGD/R treated primary astrocytes. Figure S5. The effect of knocking down circCDC14A peripherally on mRNA level of CDC14A in peripheral WBC and peri-infarct cortex. Figure S6. The effect of knocking down circCDC14A peripherally on neuroinflammation state of microglia and cytokines in tMCAO mice. [file 12974_2021_2333_MOESM1_ESM.docx]

**Additional material**

**Additional Methods**

**Primary astrocytes cultures**

Primary mouse astrocytes were isolated from postnatal (P)1 to P2 C57BL/6J mice. Mouse brains were removed quickly and placed in ice-cold PBS. After the membranes and large blood vessels were removed, the dissected brain cortices were placed in DMEM (Corning, 10–013-CVR) and then digested with trypsin-EDTA (Gibco, 25,200,056). Afterwards, the cells were planted on poly-Llysine precoated cell culture flasks containing DMEM supplemented with fetal bovine serum (FBS, 10% v: v; Gibco, 10,099–141) and penicillin/streptomycin (1% v/v, Gibco, 10,378–016). Astrocytes were cultured in a humidified incubator (37°C, 5% CO2). After 7 to 10 days, the astrocytes were harvested.

**Enzyme-linked immunosorbent assay (ELISA)**

The levels of tumour necrosis factor alpha (TNF-α), interleukin-10 (IL-10) and interleukin-6 (IL-6) in peri-infarct cortex were detected using ELISA kits (no. E-EL-M3063, E-EL-M0046c and E-EL-M0044c, Elabscience.) according to the manufacturer’s instructions.

**Western-blot**

Mice were sacrificed, and the brain tissues in peri-infarct cortex were rapidly dissected after perfused by PBS. The tissues were homogenized in RIPA lysis buffer (no. P0013B, Beyotime, Shanghai, China) supplemented with PMSF (Beyotime, Shanghai, China) and phosphatase inhibitors for the detection of phosphorylated proteins. After centrifugation, total protein was extracted from the supernatant and the protein concentrations were detected using a BAC kit (Beyotime, Shanghai, China). SDS-PAGE was used to separate and transfer proteins to PVDF membranes (Millipore Co., USA). After blocking in 5% non-fat milk for two h, the PVDF membranes were incubated with the following primary antibodies at 4 °C overnight: anti-iba-1 (no. 17198, Cell Signaling Technology, 1:1000), and anti-GAPDH rabbit antibody (no. 5174, Cell Signaling Technology, USA, 1:500). The membranes were incubated with secondary antibody (1:2000, biosharp, BL003A) for one h at 37 °C. A gel imaging instrument (Tanon, Shanghai, 5200) and Image J software were used to scan and analyze the immunoblots, respectively.

**Additional Table 1. Involved experimental mice in tMCAO modeling.**

| Experiment | Total | Death/ Failed | Mice in every group |
| --- | --- | --- | --- |
| qPCR detection of circCDC14A | 54 | 8/4 | n=6 for sham, 12h, 24h, 3d, 5d, 7d and 9d. |
| FISH | 24 | 3/3 | n=3 for sham, 1d, 2d, 3d, 5d and 7d. |
| Microinjection of lentivirus into lateral ventricle | | | |
| TTC | 24 | 4/4 | n=8/group (circCon and si-circCDC14A) |
| mNSS and survival rate analysis | 60 | 22/7 | n=28 and 25 respectively for circCon and circCDC14A group |
| Intravenously injection of lentivirus | | | |
| TTC | 24 | 5/3 | n=8/group (circCon and si-circCDC14A) |
| mNSS and survival rate analysis | 70 | 22/10 | n=30/group (circCon and si-circCDC14A) |
| Immunofluorescence staining | 40 | 6/4 | n=15/group (circCon and si-circCDC14A) |
| Western-blot and Elisa | 16 | 2/2 | n=8/group (circCon and si-circCDC14A) |

**Additional Figure 1**


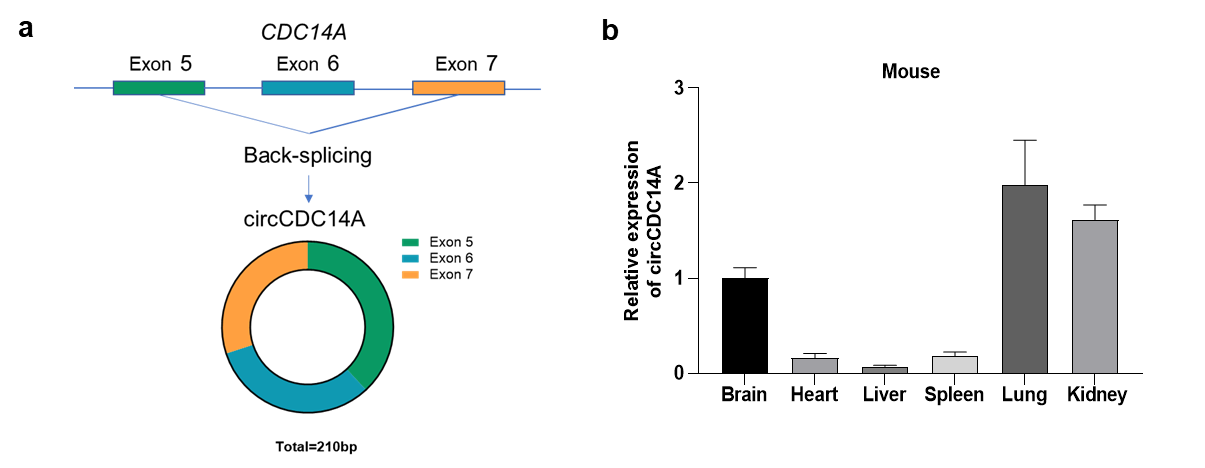


**Additional Figure 1. Gene information and organ distribution in mice of circCDC14A.** **(a)** Gene information of circCDC14A. **(b)** Relative expression of circCDC14A in brain, heart, liver, spleen, lung and kidney of mice.

Additional Figure 2


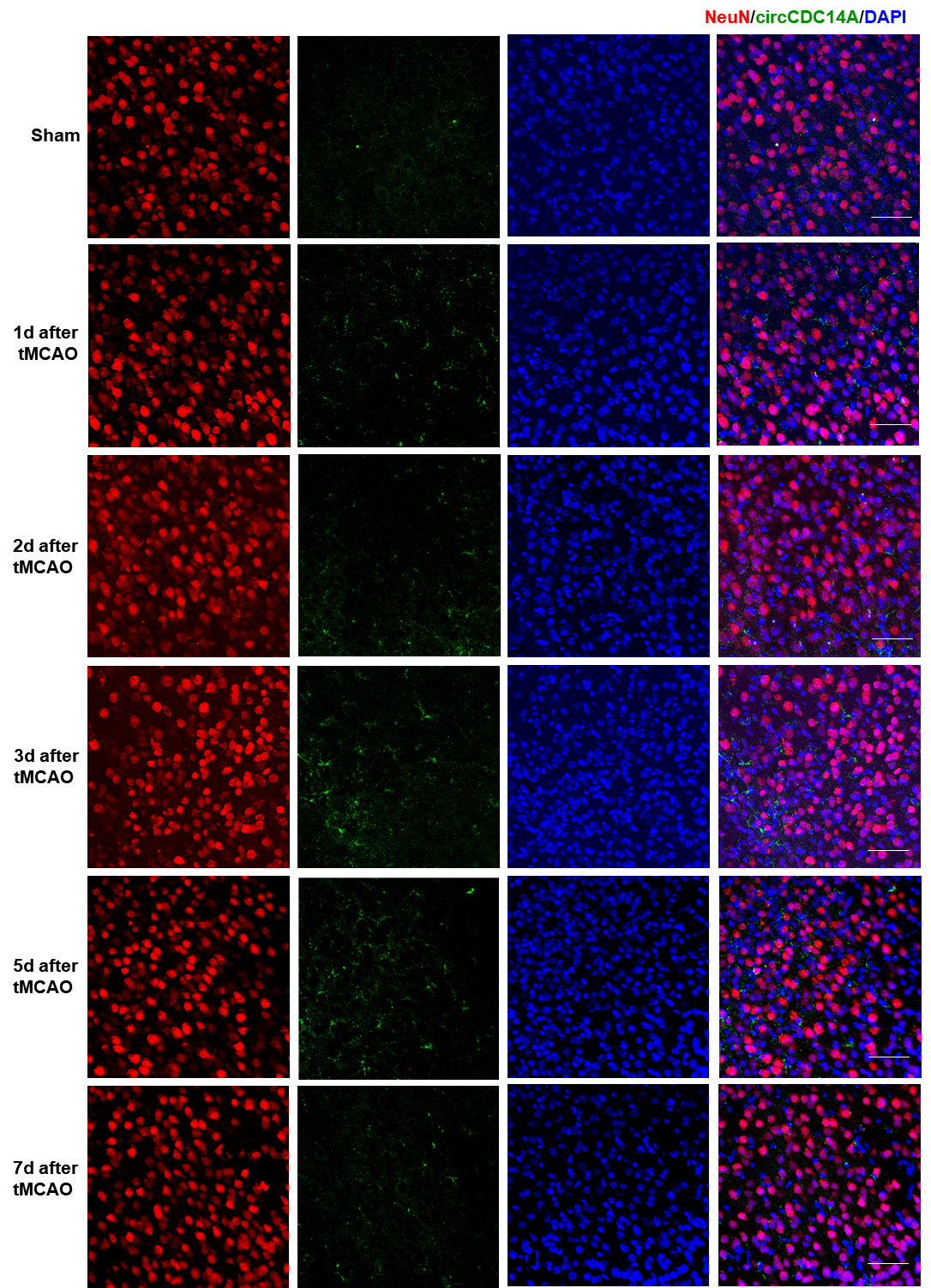


**Additional Figure 2.** **Colocalization of circCDC14A with neuron in peri-infarct cortex of sham operated and tMCAO mice from 1 day up to 7 days after modeling.** Bar:50 μm.

Additional Figure 3
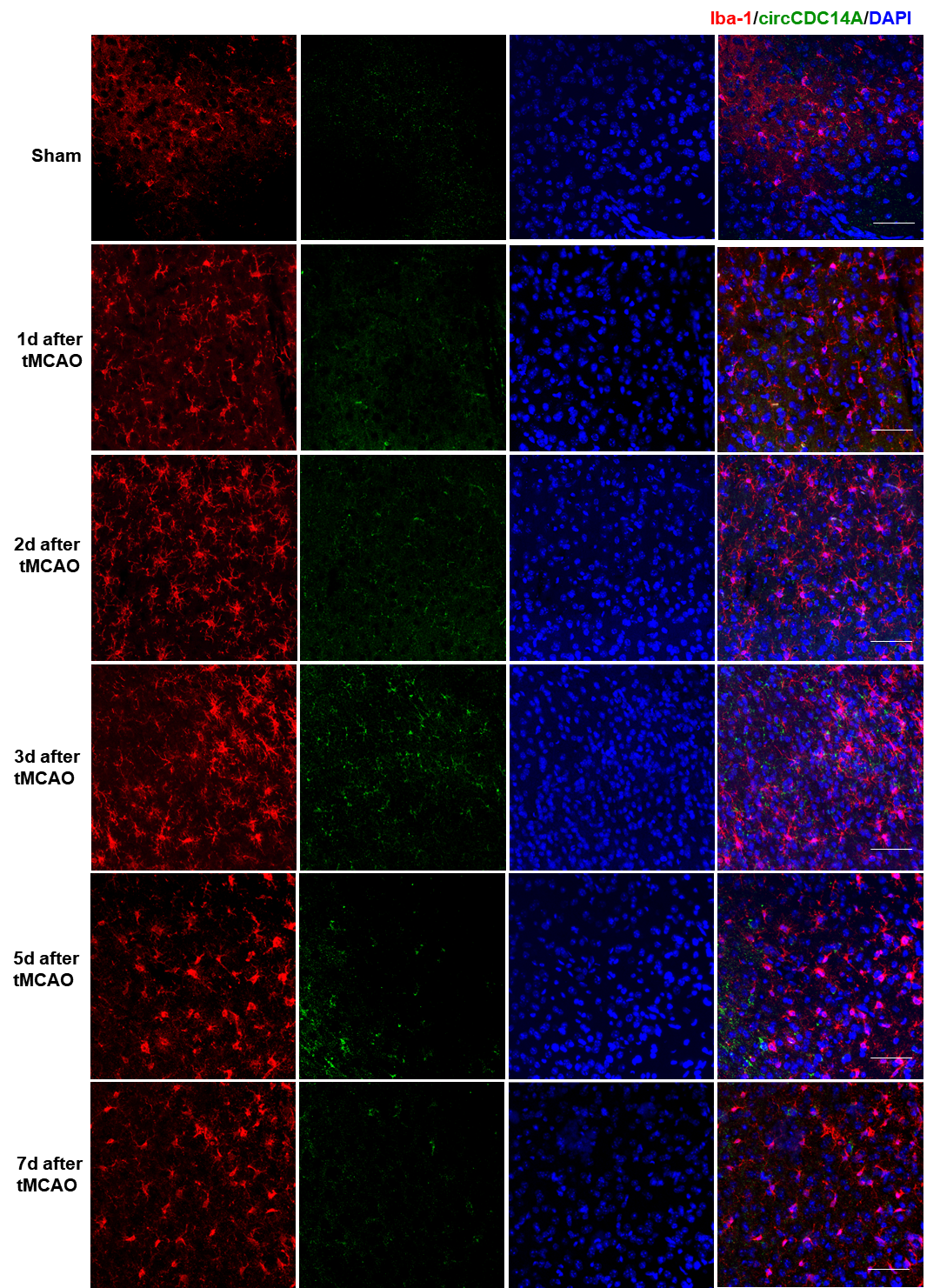


**Additional Figure 3. Colocalization of circCDC14A with microglia in peri-infarct cortex of sham operated and tMCAO mice from 1 day up to 7 days after modeling.** Bar: 50 μm.


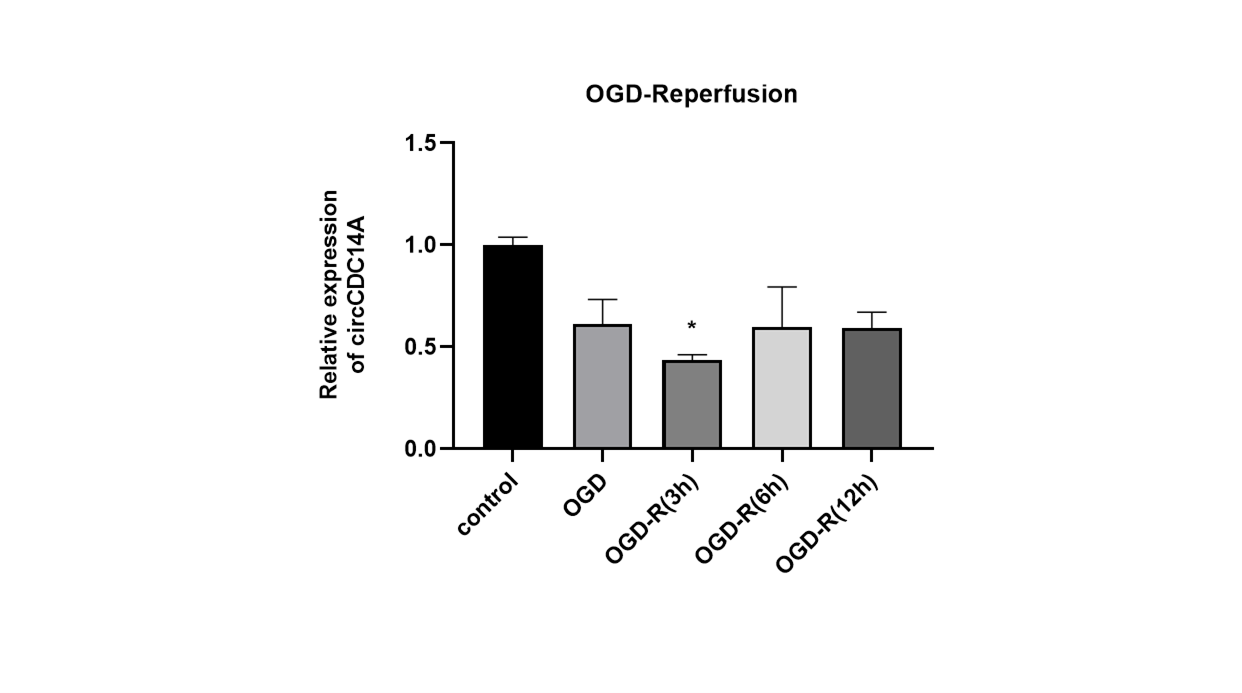
Additional Figure 4

**Additional Figure 4.** **Relative expression of circCDC14A in OGD/R treated primary astrocytes.** n=3/group, ^*^*P*＜0.05, one-way ANOVA test.

Additional Figure 5


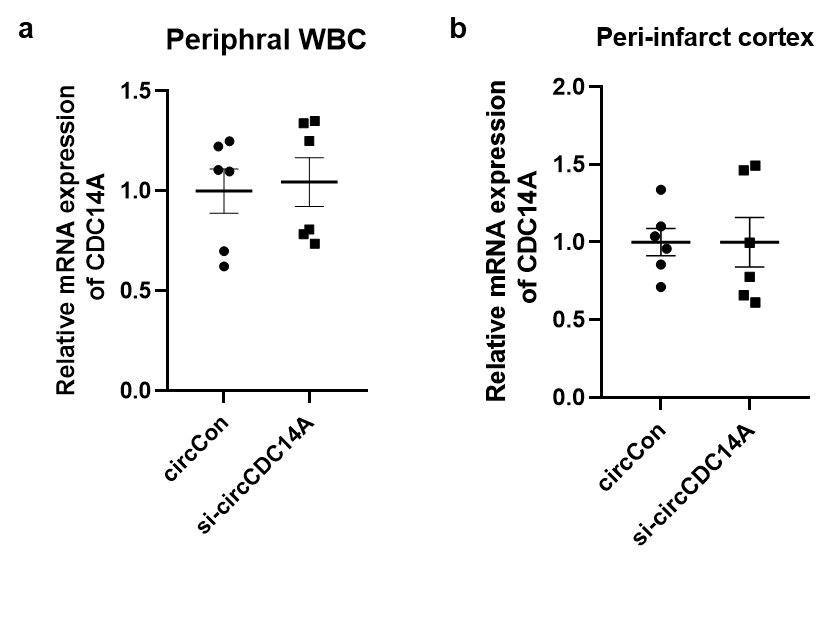


**Additional Figure 5.** **The effect of knocking down circCDC14A peripherally on mRNA level of CDC14A in peripheral WBC and peri-infarct cortex.** n=6/group, no significance, Student’s t test.

Additional Figure 6


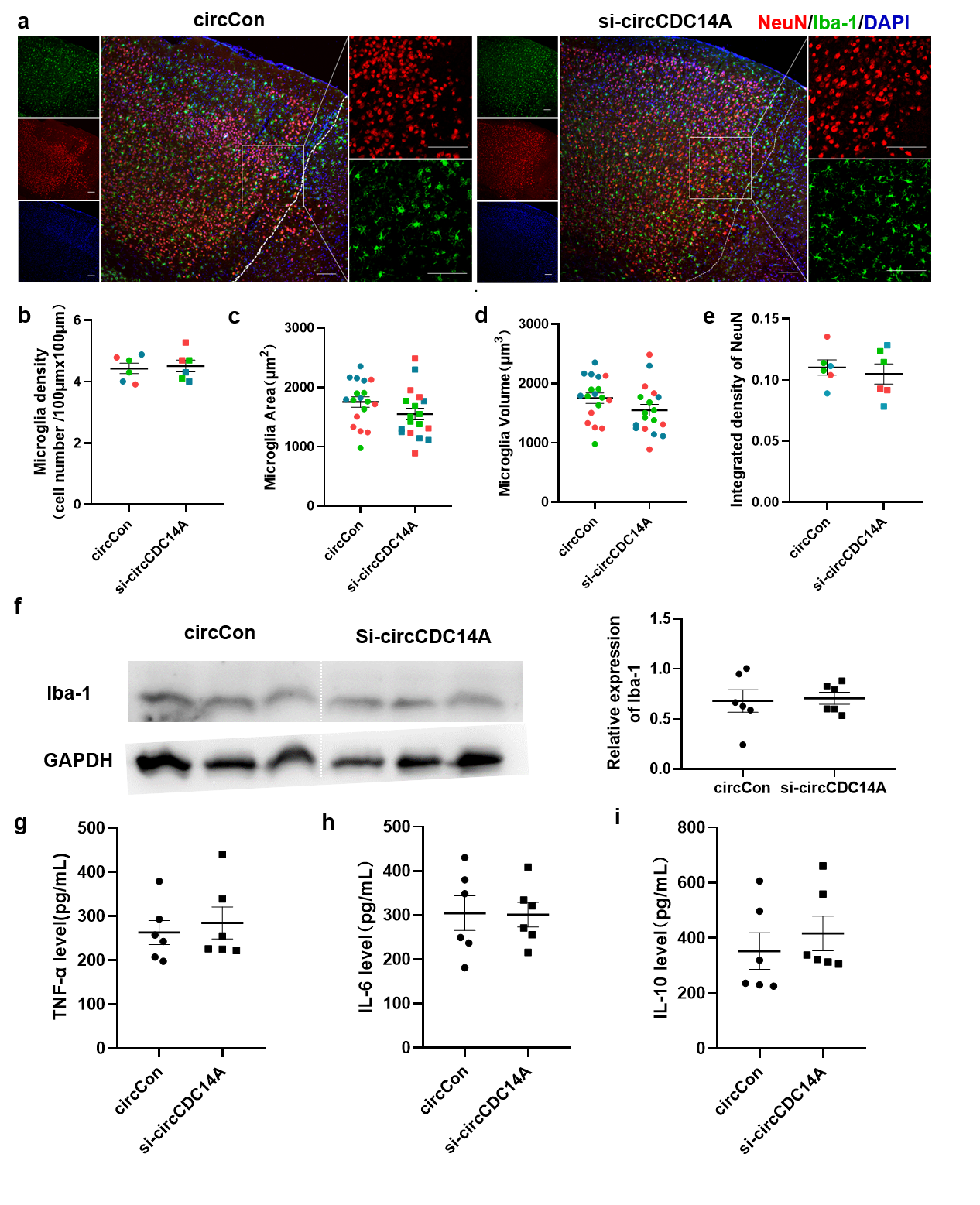


**Additional Figure 6.** **The effect of knocking down circCDC14A peripherally on neuroinflammation state of microglia and cytokines in tMCAO mice. (a)** Representative images of immunofluorescence showing activated microglia (Iba-1) and neuron (NeuN) in peri-infarct cortex 3 days after modeling. Dashed line showed the border of the infarct lesion. Bar: 100 μm. **(b)**Density of activated astrocytes in peri-infarct cortex were analyzed, n=2 field (320μm×320μm) *3 different mice represented by different colors per group, Student’s t test. **(c)** Areas and **(d)** volumes of microglia in peri-infarct cortex were analyzed by Imaris software, n=6 astrocytes (320μm×320μm) *3 different mice represented by different colors per group, Student’s t test. **(e)**Integrated immunofluorescence density of NenN^+^ were quantified, n=2 field *3 different mice represented by different colors per group, Student’s t test. **(f)**Iba-1 protein level in cirCon group and si-circCDC14A group, n=6/group, Student’s t test. **(g-i)** The level of TNF-α, IL-6 and IL-10 in peri-infarct cortex 3 days after modeling were detected, n=6/group, Student’s t test**.**
